# The CDK inhibitor AT7519 inhibits human glioblastoma cell growth by inducing apoptosis, pyroptosis and cell cycle arrest

**DOI:** 10.1038/s41419-022-05528-8

**Published:** 2023-01-09

**Authors:** Wenpeng Zhao, Liang Zhang, Yaya Zhang, Zhengye Jiang, Hanwen Lu, Yuanyuan Xie, Wanhong Han, Wentao Zhao, Jiawei He, Zhongjie Shi, Huiying Yang, Junjie Chen, Sifang Chen, Zhangyu Li, Jianyao Mao, Liwei Zhou, Xin Gao, Wenhua Li, Guowei Tan, Bingchang Zhang, Zhanxiang Wang

**Affiliations:** 1 Department of Neurosurgery and Department of Neuroscience, Fujian Key Laboratory of Brain Tumors Diagnosis and Precision Treatment, Xiamen Key Laboratory of Brain Center, the First Affiliated Hospital of Xiamen University, School of Medicine, Xiamen University, Xiamen, 361102 China; 2 Department of Medical Oncology, the First Affiliated Hospital of Xiamen University, Xiamen, 361003 China; 3 State Key Laboratory of Cellular Stress Biology, Innovation Center for Cell Signaling Network, School of Life Sciences, Xiamen University, Xiamen, Fujian 361102 China; 4 Analysis and Measurement Center, School of Pharmaceutical Sciences, Xiamen University, Xiamen, 361001 P. R. China

**Keywords:** CNS cancer, High-throughput screening, Cell death

## Abstract

Glioblastoma multiforme (GBM) is the most lethal primary brain tumor with a poor median survival of less than 15 months. However, clinical strategies and effective therapies are limited. Here, we found that the second-generation small molecule multi-CDK inhibitor AT7519 is a potential drug for GBM treatment according to high-throughput screening via the Approved Drug Library and Clinical Compound Library (2718 compounds). We found that AT7519 significantly inhibited the cell viability and proliferation of U87MG, U251, and patient-derived primary GBM cells in a dose-dependent manner. Furthermore, AT7519 also inhibited the phosphorylation of CDK1/2 and arrested the cell cycle at the G1-S and G2-M phases. More importantly, AT7519 induced intrinsic apoptosis and pyroptosis via caspase-3-mediated cleavage of gasdermin E (GSDME). In the glioblastoma intracranial and subcutaneous xenograft assays, tumor volume was significantly reduced after treatment with AT7519. In summary, AT7519 induces cell death through multiple pathways and inhibits glioblastoma growth, indicating that AT7519 is a potential chemical available for GBM treatment.

## Introduction

Glioblastoma multiforme (GBM) is the most common brain tumor with the occurrence up to ~50% in adults, which brings tremendous challenges to clinical treatment due to the complicated microenvironment of tumor cells and the intratumoral heterogeneity [1–3]. Currently, the clinical strategies for GBM treatment are radiotherapy and chemotherapy (temozolomide, TMZ) after surgical resection, but the overall prognosis is poor with a median survival of <15 months [4]. Meanwhile, the drug resistance of TMZ is the most intractable problem [5]. Thus, new drugs urgently need to be developed for GBM treatment or combined therapies.

Various drugs targeting cyclin-dependent kinases (CDKs) are widely used because the uncontrolled proliferation and dysregulation of the cell cycle are hallmarks of tumor cells [6–9]. Nowadays, three CDK inhibitors, palbociclib, ribociclib, and abemaciclib have been approved by the FDA for the treatment of advanced breast cancer [10–12]. Despite the therapeutic effect on the GBM patient-derived xenograft tumor, Roscovitine, the representative of first-generation CDK inhibitor, is not approved and widely used to clinical treatment because of the toxic effect [13]. CDK4/6 inhibitors have shown positive results in preclinical studies of glioblastoma, but clinical trials of these CDK inhibitors in glioma patients have not been satisfactory [14, 15]. As one of the second-generation CDK inhibitors, AT7519 selectively inhibits CDK1, 2, 4, 6, and 9 and shows better safety in both hematologic and solid malignancies compared to the first-generation CDK inhibitors [16–18]. AT7519 exhibits strong antitumor activity in a variety of malignant tumors, such as pancreatic cancer, lung cancer, and nasopharyngeal carcinoma [19–21]. However, the anti-glioblastoma activity and the detailed mechanism of AT7519 have never been elucidated.

In this work, we performed screening assays and found that AT7519 is a potential drug exerting antitumor activity in GBM through multiple pathways: 1) AT7519 directly targeted on CDKs to arrest the cell cycle at the G1 and G2 phases and inhibit GBM cell proliferation; 2) AT7519 induced GBM cell apoptosis through the intrinsic pathway; 3) AT7519 stimulated pyroptosis by caspase-3-mediated activation of GSDME-N. Previous studies provided evidence that apoptosis and pyroptosis were recognized as important mechanisms of classical chemotherapy drugs in tumor cells [22–25]. Pyroptosis was gasdermin-mediated cell death, including gasdermin A, B, C, D, and E [26–29]. Remarkably, a risk model of GBM was constructed based on four pyroptosis-associated genes, which have been shown as independent prognostic factors for GBM patients [30]. Thus, activation of cell apoptosis and pyroptosis by chemical drugs is feasible for GBM treatment. Our work reveals that AT7519 induces apoptosis and pyroptosis, and exhibits antitumor activity in intracranial and subcutaneous xenografted nude mice, indicating that AT7519 represents a potentially effective small molecule targeted drug for the treatment of GBM.

## Materials and methods

### Cell Culture

Human glioblastoma cell lines (U87MG and U251) were purchased from ATCC. Both cells were cultured in Dulbecco’s modified Eagle’s medium (DMEM, Gibco, Carlsbad, USA) containing 10% heat-inactivated fetal bovine serum (FBS, ABW, Shanghai, China) and 1% penicillin/streptomycin (Gibco) at 37 °C in a humidified atmosphere of 5% CO_2_. Patient-derived primary GBM cell (GBM38 and GBM60) isolation and culture were performed according to the method described by Xiang et al. [31]. Tumor tissues were obtained with the informed consent of patients and approved by the Ethics Committee of the First Affiliated Hospital of Xiamen University.

### Drugs and caspase inhibitor

The Approved Drug Library (L1000), Clinical Compound Library (L3400), and CDK inhibitor AT7519 (T6205) were purchased from Targetmol. The pancaspase inhibitor zVAD-FMK was purchased from GlpBio (GC12861). The caspase-3-specific inhibitor zDEVD-FMK was purchased from MedChemExpress (HY-12466).

### Cell proliferation assay

Cells were seeded in 96-well plates (8000 cells/well). After the cell adhesion, the medium was replaced with fresh medium containing DMSO (control) or different concentrations of AT7519 for 48 h. Then, the cells were incubated with the medium containing 10% Cell Counting Kit-8 reagent (CCK-8, K1018, ApexBio, Houston, TX, USA) for 1 h at 37 °C. The OD value was measured using a microplate reader at 450 nm. The IC_50_ values were calculated using GraphPad Prism 8.

### Colony formation assay

Cells were seeded into 6-well plates (800 cells/well) and then treated with different concentrations of AT7519 for 14 days. These cells were fixed with 4% paraformaldehyde and stained with 0.5% crystal violet for 20 min.

### EdU-DNA synthesis assay

Different concentrations of AT7519 were added to U251 and U87MG cells in the logarithmic growth phase for 48 h. The EdU Apollo 567 in vitro imaging kit (C10310, RiboBio, Guangzhou, China) was used in accordance with the manufacturer’s protocol. The cells were observed and photographed with a fluorescence microscope after EdU staining.

### RNA sequencing and gene enrichment analysis

U87MG cells were incubated with 0.4 μM AT7519 for 48 h and lysed in TRIzol reagent. The total RNA quantity and purity were assessed using a Bioanalyzer 2100 and RNA 6000 Nano LabChip Kit (Agilent, CA, USA, 5067-1511), and high-quality RNA samples with RIN > 7.0 were used to construct the sequencing library. After building the library, we performed 2 × 150 bp paired-end sequencing (PE150) on an Illumina Novaseq™ 6000 following the vendor’s recommended protocol.

### Cell cycle and apoptosis assay

U251 and U87MG cells were treated with DMSO (0.1%) and 0.4 μM AT7519 for 6, 12, and 24 h. Cells were collected and fixed overnight with 70% ice-cold ethanol. Then, 500 μL PI/RNase A working solution containing 50 μg/mL PI (P4170, Sigma Aldrich, St. Louis, MO, USA), and 100 μg/mL RNaseA (10109142001, Roche Diagnostics, Mannheim, Germany) was added. The cell cycle was detected by flow cytometry. After the cells were stained with reagents from the PI/Annexin V-FITC kit (556547, BD Biosciences, Franklin Lakes, NJ, USA), cell apoptosis was detected by flow cytometry.

### JC-1 assay

Cells were seeded in 6-well plates and treated with 0.4 µM AT7519 for 48 h. The Mitochondrial membrane potential assay kit (C2006, Beyotime, Shanghai, China) was used in accordance with the manufacturer’s protocol. The cell images were captured under the fluorescence microscope immediately. Fluorescence intensity was measured by spectrofluorimeter.

### Scanning electron microscopy (SEM)

GBM cells were fixed in PBS containing 2.5% glutaraldehyde for 2 h and washed three times with PBS for 15 min. Then, cells were dehydrated by increasing concentrations of ethanol (30%, 40%, 50%, 60%, 70%, 80%, 90% and 100%). After drying in the critical point dryer, the cells were sprayed by ion sputter. Finally, the sample was observed by SEM (Hitachi SU8100).

### Lactate dehydrogenase (LDH) release assay

A cytotoxicity LDH assay kit (c0017, Beyotime, Shanghai, China) was used to detect the level of LDH released by cells. After centrifugation, the supernatant was collected, and the experiment was performed according to the manufacturer’s instructions. The percentage of LDH release was calculated using the equation (LDH sample-LDH background)/(LDH maximum-LDH background) × 100%.

### Protein detection by Western blotting

Western blot analysis of GBM cells treated with AT7519 was performed as described previously [32]. Antibodies against p-RB (T821) (ab32015), RB (ab181616), and GSDME (ab215191) were purchased from Abcam. Antibodies against p-CDK1 (T161) (9114), CDK1 (9116), Cyclin B1 (12231), Caspase-3 (14220), Cleaved Caspase-3 (9661), Cleaved PARP (5625), Caspase-9 (9502), MCL-1 (94296), and BCL-2 (15071) were purchased from Cell Signaling Technology. Antibodies against GAPDH (60004-1-Ig), PARP1 (13371-1-AP), p-P53 (S15) (28961-1-AP), P53 (10442-1-AP), and Cyt-c (10993-1-AP) were purchased from Proteintech. Antibodies against p-CDK2 (T160) (AP0325), CDK2 (A0094), Cyclin E1 (A12000), and NOXA (A9801) were purchased from ABclonal.

### Immunohistochemistry

Tumor tissue was fixed with 4% paraformaldehyde, embedded in paraffin and sectioned. Immunohistochemical staining was performed as described previously [33]. Images were captured with an EVOS M7000 microscope.

### Subcutaneous xenograft model

All animal experiments were reviewed and approved by the Animal Ethics Committee of Xiamen University. Female nude mice (BALB/c nude, 6 weeks old) were obtained from Xiamen University Animal Experiment Center and housed at the animal barrier facility of Xiamen University School of Medicine. U87MG cells (5 × 10^6^) were injected subcutaneously into nude mice. Five days later, the mice were randomly divided into two groups (*n* = 6) and treated with AT7519 once daily for 3 weeks (20 mg/kg, i.p.). The body weights of the nude mice were recorded every 5 days. In addition, we measured tumor volume once a week. Tumor volume was calculated using the following formula: *V* = *L* × *W*^2^ × 1/2 (*V*, volume; *L*, length; *W*, width). At the end of the experiment, the mice were euthanized. The tumor was removed and weighed.

### Intracranial xenograft model

After nude mice were anesthetized by isoflurane inhalation, 1 × 10^5^ U87MG cells suspended in 4 μL of PBS were slowly injected into the left striatum using a 10-µL Hamilton syringe at the following coordinates related to the bregma: 1.0 mm lateral, 1.0 mm anterior, and 3.5 mm in depth. Five days later, the mice were randomly divided into two groups (*n* = 4) and treated with AT7519 once daily for 3 weeks (20 mg/kg, i.p.). After 21 days of continuous dosing, the mice were sacrificed and perfused with ice-cold PBS and 4% paraformaldehyde (PFA) from the apex of the heart. The mouse brains were subsequently dissected and fixed in 4% PFA for 24 h, and paraffin-embedded sections were HE stained.

### Statistical analysis

All experiments were repeated at least thrice, and the experimental data were statistically analyzed using GraphPad Prism 8.0 software. Data are presented as the means ± standard deviations (SD). An unpaired *t*-test was used for comparisons between two groups, and one-way ANOVA was used for comparisons between multiple groups.

## Results

### AT7519 inhibits GBM cell viability and proliferation

**Fig. 1:**
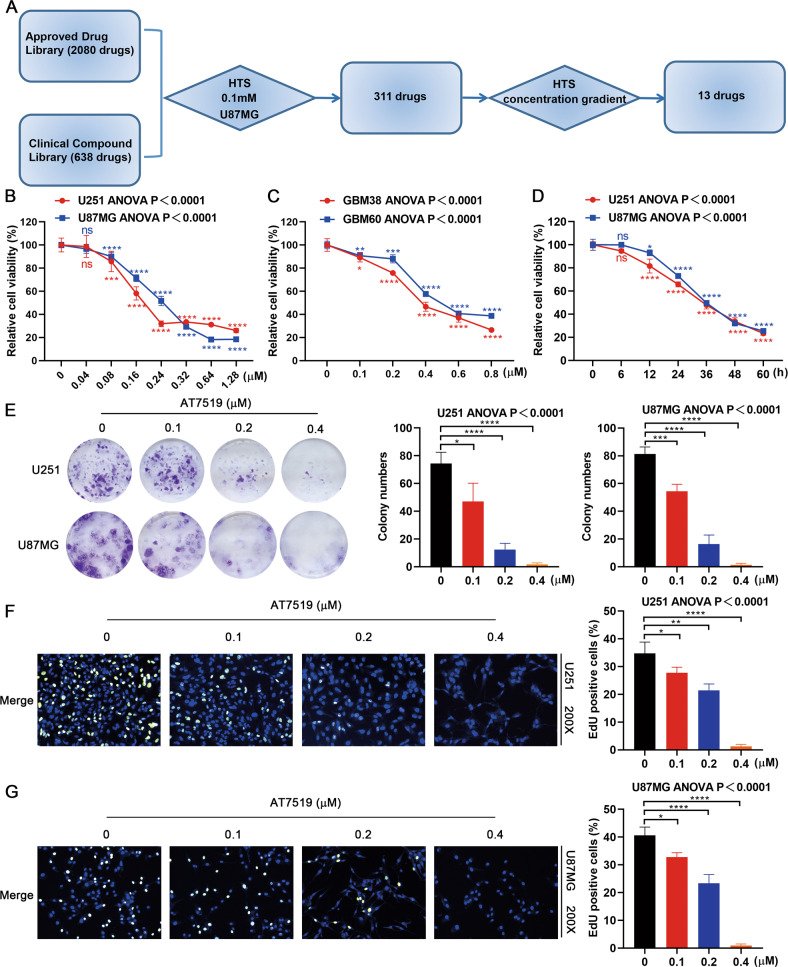
AT7519 inhibits glioblastoma cell proliferation and viability in vitro. **A** Process for high-throughput drug screening. **B**, **C** U87MG, U251, GBM60, and GBM38 cell viability was determined using a CCK-8 assay after treatment with various concentrations of AT7519. *P < 0.05, ***P < 0.01, ***P < 0.001, and ****P < 0.0001 compared with the control using one-way ANOVA followed by Dunnett’s multiple test. **D** U87MG and U251 cell viability was determined using a CCK-8 assay after treatment with 0.4 µM AT7519 for 6, 12, 24, 48, and 60 h. *P < 0.05, ****P < 0.0001 compared with the control using one-way ANOVA followed by Dunnett’s multiple test. **E** Colony formation assays to verify the effect of AT7519 on glioblastoma cell proliferation. *P < 0.05, ***P < 0.001, and ****P < 0.0001 based on one-way ANOVA followed by Dunnett’s multiple test. **F**, **G** The level of DNA synthesis of U87MG and U251 cells was determined using an EdU assay after treatment with increasing concentrations of AT7519. The nuclei were stained with Hoechst (blue), and the proliferating cells were stained with EdU (yellow). *P < 0.05, ***P < 0.001 and ****P < 0.0001 based on one-way ANOVA followed by Dunnett’s multiple test. The results are presented as the mean ± SD from three independent experiments.

To identify new drugs for the treatment of GBM, we used high-throughput screening to screen potential drugs from the Approved Drug Library and Clinical Compound Library (a total of 2718 compounds) for the treatment of GBM. According to the cell viability results, we found that 13 drugs, including AT7519, exhibited potential anti-GBM activity (Fig. 1A). CDKs are the main regulatory proteins of the cell cycle, and their expression reflects the proliferative state of the tumor. More importantly, the expression of CDKs is negatively correlated with GBM patient survival. Therefore, the CDK inhibitor AT7519 was selected to explore its anti-GBM activity. We treated U251 and U87MG cells with AT7519 and performed CCK8 experiments. The results showed that AT7519 dramatically inhibited GBM cell proliferation in a dose- and time-dependent manner (Fig. 1B, D), and the IC_50_ values of AT7519 on U251 and U87MG cells at 48 h were 0.246 μM and 0.2218 μM, respectively. We performed the same experiment in GBM primary cells. As expected, AT7519 inhibited the proliferation of GBM primary cells in a dose-dependent manner (Fig. 1C). The results of plate cloning experiments showed that the number of clones formed by U251 and U87MG cells substantially decreased with increasing AT7519 concentration (*P* < 0.0001, one-way ANOVA) (Fig. 1E). The EdU staining assay showed that AT7519 notably inhibited DNA synthesis in U251 and U87MG cells in a dose-dependent manner (*P* < 0.0001, one-way ANOVA) (Fig. 1F, G). The above results demonstrated that AT7519 could inhibit GBM cell activity and proliferation.

### Differential gene expression and pathway enrichment analysis of GBM cells after AT7519 treatment

**Fig. 2:**
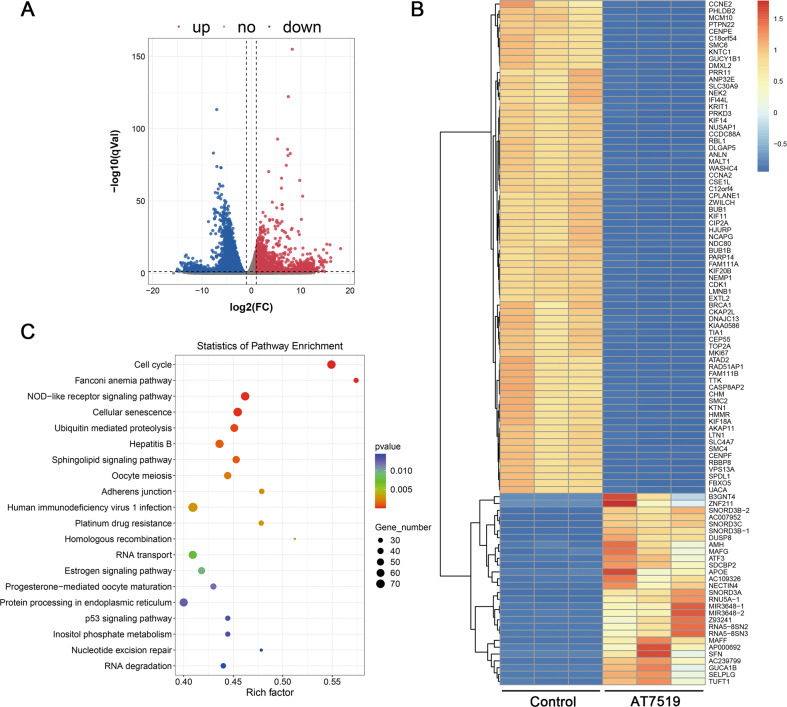
Differential gene expression and pathway enrichment analysis of glioblastoma cells treated with AT7519. **A**, **B** U87MG cells were treated with AT7519 for 48 h for RNA sequencing, and a volcano plot and heatmap of differential expression were obtained by analysis (upregulated genes are in red; downregulated genes are in blue; nonregulated genes are in gray (|log2FC | ≥ 1 and *q*-value ≤ 0.05). **C** KEGG pathway analysis of differentially expressed genes. The volcano map was drawn based on R (https://www.r-project.org/) on the OmicStudio platform (https://www.omicstudio.cn/tool). KEGG pathway analyses were performed using the OmicStudio tools at https://www.omicstudio.cn/tool.

U87MG cells were treated with AT7519 for 48 h, and RNA was extracted for RNA sequencing. The results showed that compared with the control group, there were 2185 upregulated genes and 5112 downregulated genes in the AT7519-treated group (|log2FC | ≥ 1 and *q* < 0.05) (Fig. 2A, B). The KEGG pathway enrichment analysis of U87MG cells after AT7519 treatment mainly focused on the cell cycle, DNA damage repair, NOD-like receptors, p53, and other signaling pathways (Fig. 2C). The above results provide a direction for exploring the specific mechanism of AT7519 in GBM cells.

### AT7519 arrests the cell cycle at the G1/S and G2/M phases in GBM cells

**Fig. 3:**
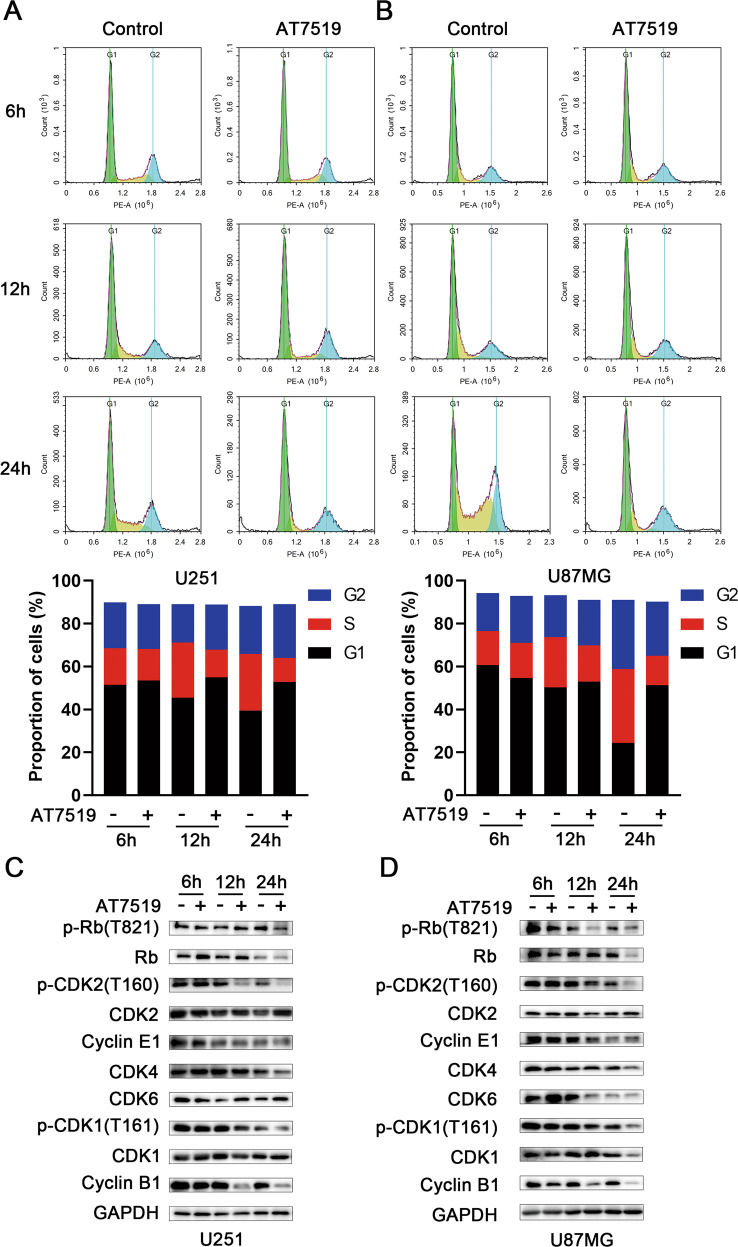
AT7519 arrests glioblastoma cells at the G1/S and G2/M phases of the cell cycle. **A**, **B** After U87MG and U251 cells were treated with different concentrations of AT7519 for 48 h, the cell cycle distribution was detected by flow cytometry. **C**, **D** Western blot analysis of cell lysates treated with different concentrations of AT7519 for 48 h. Detection of G1/S and G2/M arrest-related proteins.

After cell cycle synchronization with serum deprivation, U251 and U87MG cells were cultured with media alone and AT7519 (0.4 μM) for 6, 12 and 24 h. Flow cytometric analysis of cell cycle distribution showed that the proportion of G1 and G2 phase U251 and U87MG cells were increased as early as 6 h after AT7519 treatment. After AT7519 treatment of U251 and U87MG cells for 24 h, the proportion of cells in G1 phase increased significantly (Fig. 3A, B). Western blot analysis of key protein molecules related to the cell cycle showed that the expression levels of p-RB and p-CDK2, which are related to the G1-S transition of the cell cycle, were significantly decreased, and the expression levels of p-CDK1 and cyclin B1, which are related to the G2-M transition of the cell cycle, were significantly decreased (Fig. 3C, D). Taken together, AT7519 arrested U251 and U87MG cells in the G1 and G2 phases.

### AT7519 induces caspase-dependent apoptosis in GBM cells

**Fig. 4:**
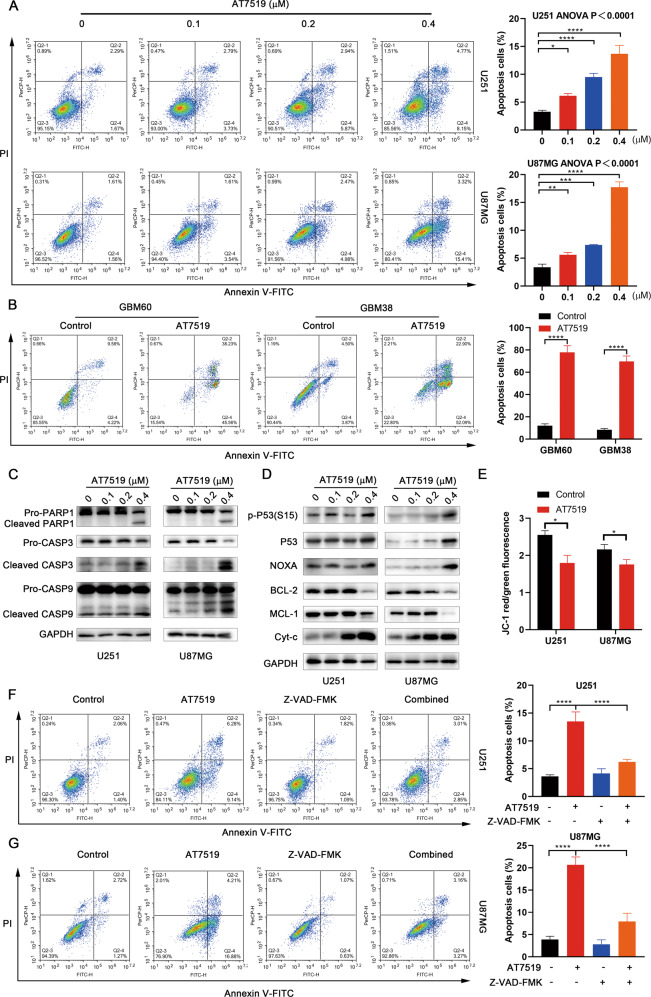
AT7519 induces apoptosis in glioblastoma cells. **A**, **B** After treatment of U87MG, U251, and GBM-derived primary cells with AT7519 for 48 h, the percentage of cells undergoing apoptosis was detected by flow cytometry using PI/Annexin V-FITC double staining. *P < 0.05, **P < 0.01, ***P < 0.001, and ****P < 0.0001 as determined by one-way ANOVA followed by Dunnett’s multiple test. **C**, **D** Western blotting detection of proteins associated with apoptosis in U87MG and U251 cells treated with 0.1, 0.2, or 0.4 μM AT7519 for 48 h. **E** Mitochondrial membrane potential was displayed by change in the ratio between red (aggregated JC-1) and green (monomeric JC-1) fluorescence intensity measured by spectrofluorimeter. *P < 0.05 by Student’s *t*-test. **F**, **G** U87MG and U251 cells were treated with AT7519 following 2-h pretreatment with Z-VAD-FMK or DMSO, and the apoptosis ratio was detected by flow cytometry using PI/Annexin V-FITC double stain. ****P < 0.0001 as determined by Student’s *t*-test.

After incubation with AT7519, the cells were stained with PI and Annexin-V-FITC. Flow cytometry results showed that AT7519 observably increased the percentages of apoptotic U251 and U87MG cells in a dose-dependent manner (*p* < 0.0001, one-way ANOVA) (Fig. 4A). The percentages of apoptotic cells with or without AT7519 treatment were 12.03% versus 77.93% (*p* < 0.0001, unpaired *t*-test) in GBM60 cells and 8.39% versus 69.73% in GBM38 cells (*p* < 0.0001, unpaired *t*-test), respectively (Fig. 4B). Western blot results showed that as the AT7519 concentration increased in U251 and U87MG cells, the expression levels of the antiapoptotic proteins Mcl-1 and BCL-2 significantly decreased, whereas the expression levels of the proapoptotic protein NOXA and mitochondrial-released cytochrome c (Cyt c) were significantly increased (Fig. 4D). These endogenous apoptosis signaling molecules coincided with activation of downstream caspase-9, caspase-3, and PARP1, as evidenced by the accumulation of cleaved caspase-9, cleaved caspase-3, and cleaved PARP1 (Fig. 4C). In addition, we also found that P53 and p-P53 protein expression increased (Fig. 4D), indicating that the P53 cell signaling pathway was activated. The results of JC-1 staining showed that AT7519 observably reduced the mitochondrial membrane potential in U251 and U87MG cells (*p* < 0.05, unpaired *t*-test) (Fig. 4E, Supplementary Fig. 1A). The expression of key apoptosis proteins was further detected in GBM primary cells. As expected, the hallmark proteins of apoptosis, cleaved PARP1 and cleaved caspase-3, were significantly increased in the AT7519-treated group (Supplementary Fig. 1B). The above data suggested that AT7519 could induce apoptosis of GBM cells. After pretreatment with the pancaspase inhibitor zVAD-FMK for 2 h, U251 and U87MG cells were incubated with AT7519 for an additional 48 h. Flow cytometry showed that zVAD-FMK treatment remarkably reduced the number of apoptotic GBM cells (*p* < 0.0001) (Fig. 4F, G). These results indicated that AT7519 induced caspase-dependent apoptosis in GBM cells.

### AT7519 induces pyroptosis through caspase-3 cleavage of gasdermin E

**Fig. 5:**
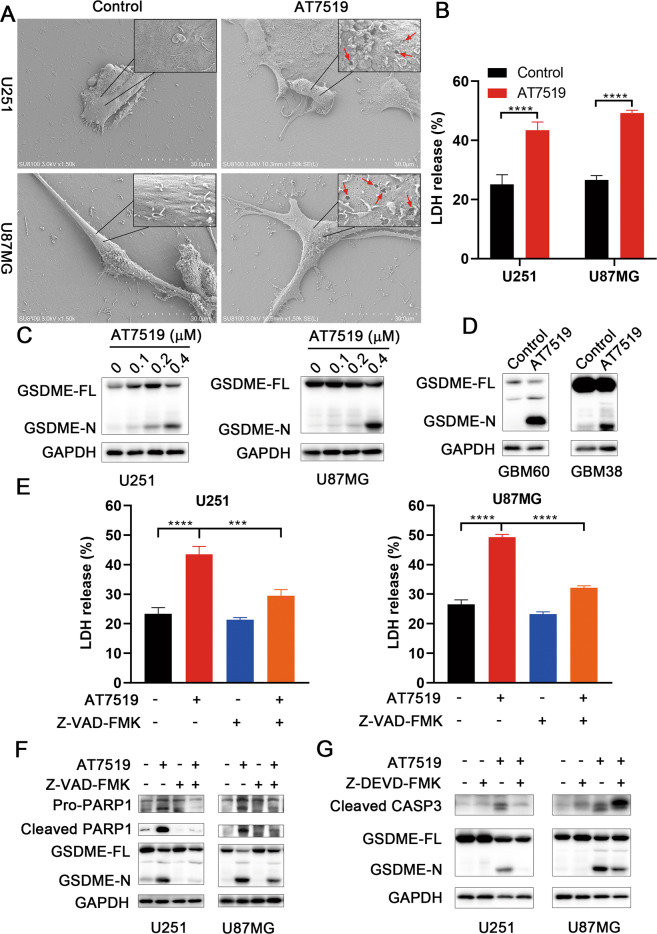
AT7519 induces pyroptosis through caspase-3 cleavage of gasdermin E. **A** U87MG and U251 cells were treated with AT7519 and morphological features of pyroptosis in SEM (red arrows, membrane pore-forming). **B** After U87MG and U251 cells were treated with AT7519 for 48 h, cytotoxicity was detected by lactate dehydrogenase (LDH) released into the cell culture medium. ****P < 0.0001 by Student’s *t*-test. **C**, **D** Full-length GSDME (GSDME-FL) and N-terminal GSDME (GSDME-N) were detected in glioblastoma cells after treatment with AT7519 for 48 h by western blot analysis. **E**, **F** U87MG and U251 cells were pretreated with Z-VAD-FMK for 2 h and then treated with AT7519 for 48 h. Cytotoxicity was detected by LDH release assay. ***P < 0.001 and ****P < 0.0001 as assessed by Student’s *t*-test. The apoptosis marker cleaved PARP and pyroptosis marker GSDME-N were detected by western blot. **G** U87MG and U251 cells were treated with Z-DEVD-FMK combined with AT7519 for 48 h, and western blot analysis of cleaved caspase-3, GSDME-FL and GSDME-N proteins was performed.

In pyroptosis, pores are formed in the cell membrane, which cause cell swelling and rupture. We first observed the morphology of GBM cells. Compared with the control group, balloon-like membrane protrusions were observed in AT7519-treated group (Supplementary Fig. 1C). SEM revealed multiple pores formed in the membranes of AT7519-treated U251 and U87MG cells (Fig. 5A). In addition, LDH release from U251 and U87MG cells was markedly increased (p < 0.0001, unpaired *t*-test) (Fig. 5B), suggesting damaged cell membrane integrity. Cleavage of gasdermin family proteins is an important hallmark of pyroptosis. Western blotting results showed that AT7519 treatment led to elevated levels of the N-terminal fragment of GSDME in U251 and U87MG cells in a dose-dependent manner (Fig. 5C). The caspase-3-specific inhibitor zDEVD‐FMK inhibited GSDME-N expression (Fig. 5G). We further verified that AT7519 could induce the cleavage of GSDME in GBM primary cells (Fig. 5D). The above results demonstrated that AT7519 produced pore-forming proteins through caspase-3 cleavage of gasdermin E, leading to cell pyroptosis. Furthermore, U251 and U87MG cells were pretreated with the pancaspase inhibitor zVAD-FMK and then treated with AT7519. We found that zVAD-FMK treatment inhibited the cleavage of GSDME (Fig. 5F) and the release of LDH (Fig. 5E) induced by AT7519.

### AT7519 inhibits tumor growth in GBM xenograft mice

**Fig. 6:**
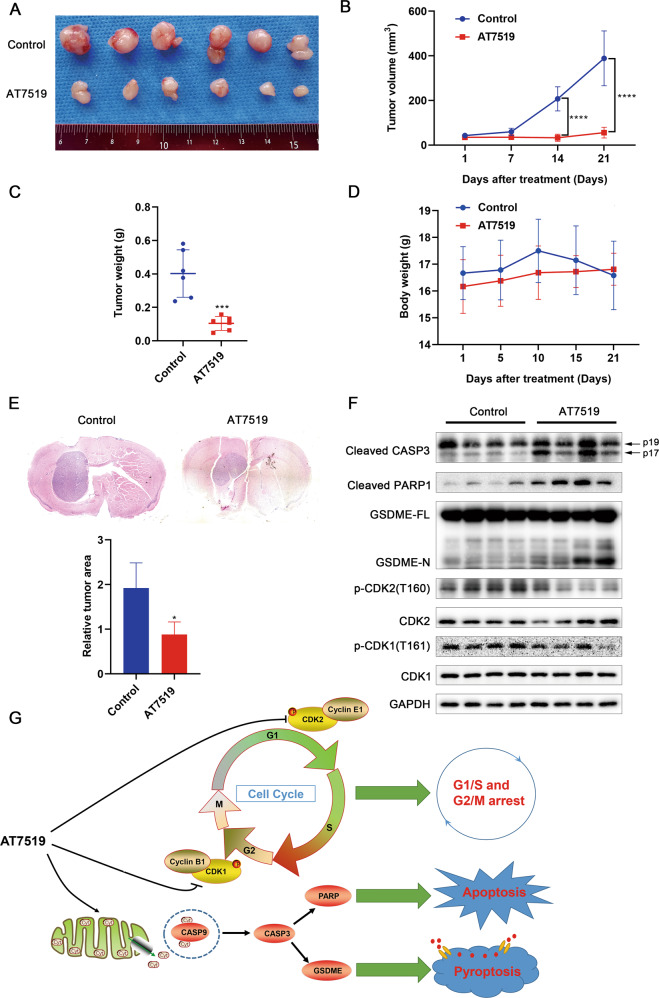
AT7519 inhibits tumor growth in glioblastoma xenograft mice. **A** Image of the subcutaneous xenograft tumors formed in nude mouse models. **B** Tumor volumes were measured and calculated every week. ****P < 0.0001 as assessed by two-way ANOVA followed by Sidak’s multiple test. **C** Tumors were excised and weighed at the end of the experiment. ***P < 0.001 by Student’s *t*-test. **D** Body weight of nude mice during administration of AT7519. **E** Representative H&E-stained images of the intracranial xenograft model in the AT7519 treatment group and control group. *p < 0.05 as assessed by Student’s *t*-test. **F** Western blot assay of the apoptosis, pyroptosis, and cell cycle-related key protein expression levels in tumor tissue. **G** Schematic model of the antitumor mechanism of AT7519 in glioblastoma cells.

To explore whether AT7519 exhibited an antitumor effect in vivo, we established a GBM subcutaneous xenograft model. The tumor volume was significantly reduced from Day 14 in nude mice injected intraperitoneally with AT7519 (*p* < 0.0001) (Fig. 6B). The tumor weight was also markedly reduced (*p* < 0.001, unpaired *t*-test) (Fig. 6C). No significant change in the body weight of nude mice was noted (Fig. 6D). After preliminary exploration of the safety and antitumor effect of AT7519 in vivo, we verified the effect of AT7519 in a GBM orthotopic xenograft model. The results of HE staining of brain tissue showed that the relative area of intracranial tumors was reduced by approximately half upon AT7519 treatment (*p* < 0.05, unpaired *t*-test) (Fig. 6E). Western blot analysed apoptosis, pyroptosis, and cell cycle-related key proteins, the results showed that increased levels of cleaved caspase-3, cleaved PARP1, and GSDME-N in the AT7519-treated group, while the levels of the p-CDK1 and p-CDK2 proteins were decreased (Fig. 6F). Immunohistochemical staining also confirmed a similar trend (Supplementary Fig. 2).The above results demonstrated that AT7519 inhibited glioblastoma growth by inducing apoptosis, pyroptosis and cell cycle arrest in vivo.

## Discussion

GBM is one of the most common and malignant tumors of the central nervous system, and the current 5-year survival rate of GBM patients remains <5% [34]. TMZ has been the only chemotherapy drug approved for the treatment of GBM. However, TMZ drug resistance and side effects are important factors that lead to poor treatment effects [35]. There is an urgent need to find new drugs for GBM. In this study, we selected AT7519 as a potential drug for the treatment of GBM according to high-throughput drug screening results via the Approved Drug Library and Clinical Compound Library, which chemicals are already in clinical research or approved for clinical usage.

Previous studies on the antitumor mechanism of AT7519 mainly focused on cell cycle arrest and apoptosis [36, 37]. Our study confirmed that AT7519 arrested the cell cycle at the G1 and G2 phases and induced intrinsic apoptosis in GBM. Furthermore, we found that AT7519 stimulates pyroptosis by caspase-3-mediated cleavage of GSDME-N. The blood-brain barrier is an important factor hindering the development of drugs in the central nervous system [38]. Our experiments found that a certain concentration of AT7519 could be detected in the brain tissue of mice after injection with AT7519. The intracranial tumors were significantly reduced after treatment with AT7519. Our work reveals that AT7519 is a potential drug for GBM treatment.

A multitude of problems need to be solved when new drugs are used for GBM treatment, including toxic effect and dug resistance. Many studies have found that apoptosis escape is an important factor affecting the efficacy of drug treatment [39, 40]. AT7519 induces pyroptosis through the cleavage of GSDME by caspase-3, suggesting that AT7519 is a potential chemical to solve the antiapoptotic drug resistance. In future, the combined treatment of AT7519 and radiotherapy or chemotherapy (TMZ) needs to be evaluated according the improvement on toxic effect and dug resistance. In conclusion, our study provided new evidence and new strategies for GBM treatment.

## Supplementary information

Supplemental Figure Legend

Supplementary Fig. 1

Supplementary Fig. 2

aj-checklist

Original Data File

Original Data File

## Data Availability

The authors declare that all data in the article is available.
