# Supplementary material for: The CDK inhibitor AT7519 inhibits human glioblastoma cell growth by inducing apoptosis, pyroptosis and cell cycle arrest
Source: Cell Death Dis. 2023 Jan 9;14(1):11. doi: 10.1038/s41419-022-05528-8 (PMC9829897; doi:10.1038/s41419-022-05528-8)
Supplement: Supplementary file 1 — Supplemental Figure Legend [file 41419_2022_5528_MOESM1_ESM.docx]

**Supplementary Fig. 1 (A**) JC-1 staining showed changes in the mitochondrial membrane potential of U87 and U251 cells cultured in the absence or presence of AT7519. **(B)** In GBM-derived primary cells, Western blotting detected the expression of the key apoptosis proteins cleaved caspase-3 and cleaved PARP1. **(C)** U87MG and U251 cells were treated with AT7519 and imaged (red arrows, pyroptotic cells).

**Supplementary Fig. 2** IHC to detect changes in the expression of [proliferation](javascript:;), apoptosis, and cell cycle-related key proteins in vivo
