# Supplementary figures and images for: The CDK inhibitor AT7519 inhibits human glioblastoma cell growth by inducing apoptosis, pyroptosis and cell cycle arrest
Source: Cell Death Dis. 2023 Jan 9;14(1):11. doi: 10.1038/s41419-022-05528-8 (PMC9829897; doi:10.1038/s41419-022-05528-8)

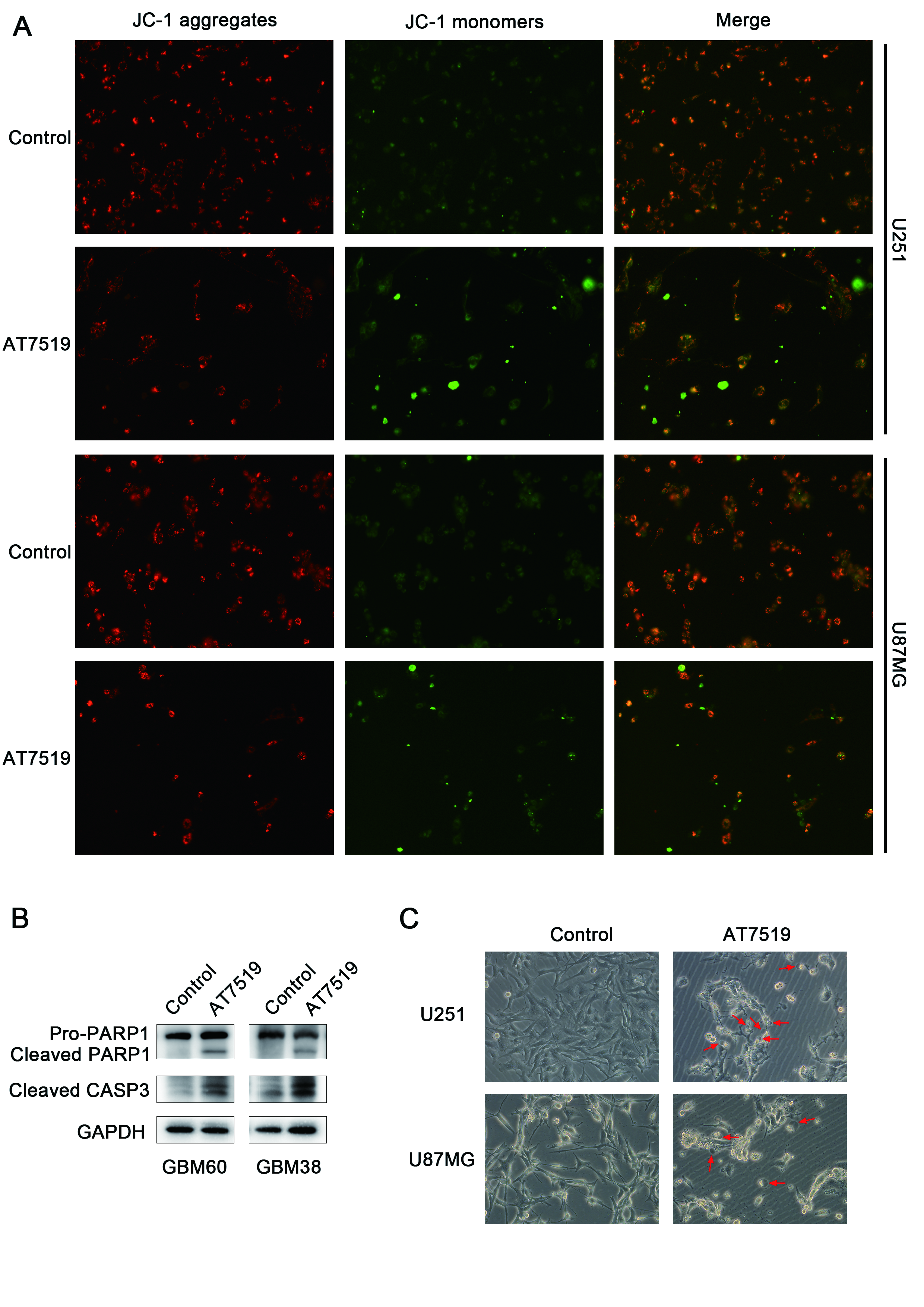

Supplement: Supplementary file 2 — Supplementary Fig. 1 [file 41419_2022_5528_MOESM2_ESM.tif]

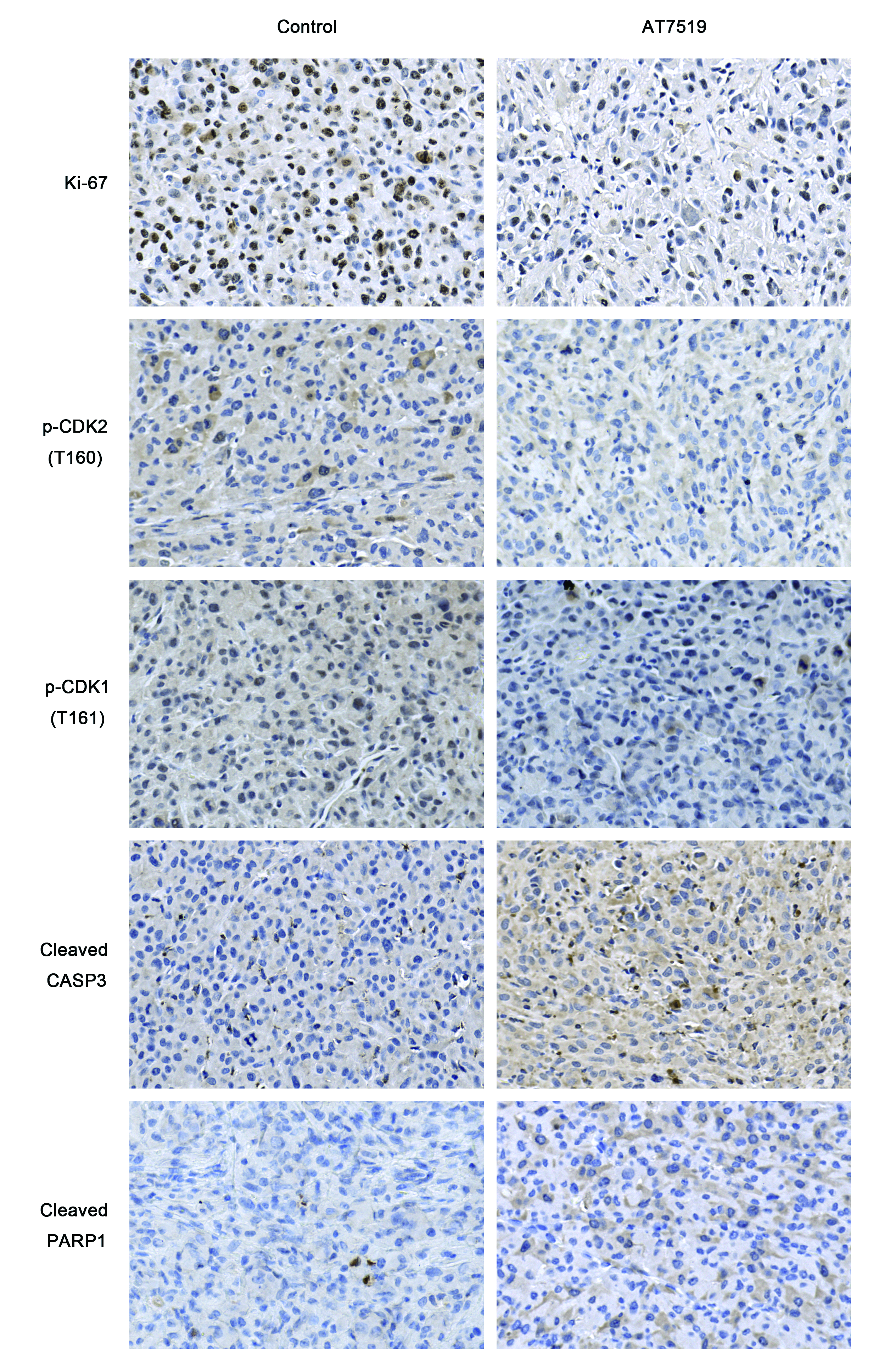

Supplement: Supplementary file 3 — Supplementary Fig. 2 [file 41419_2022_5528_MOESM3_ESM.tif]

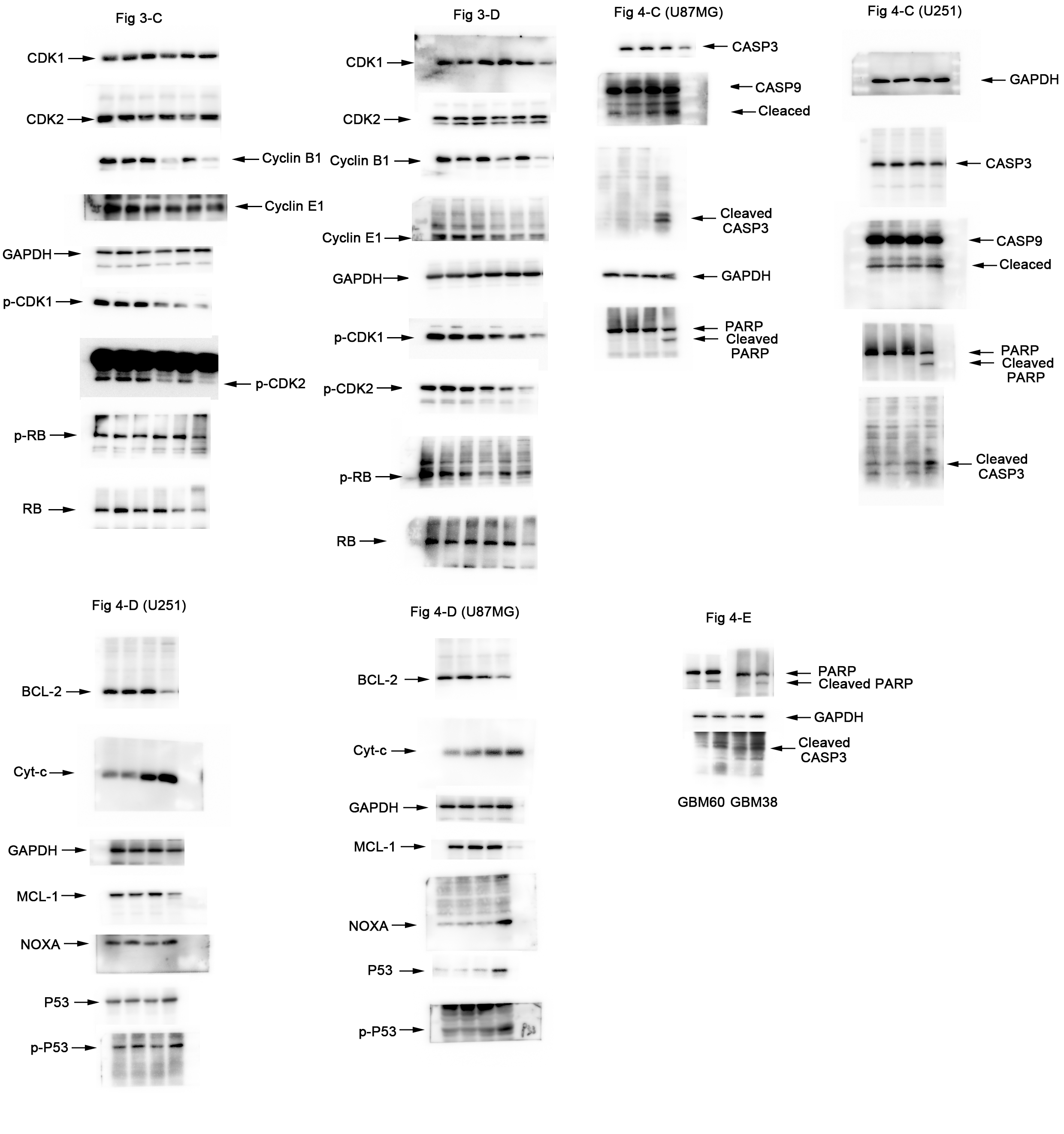

Supplement: Supplementary file 5 — Original Data File [file 41419_2022_5528_MOESM5_ESM.tif]

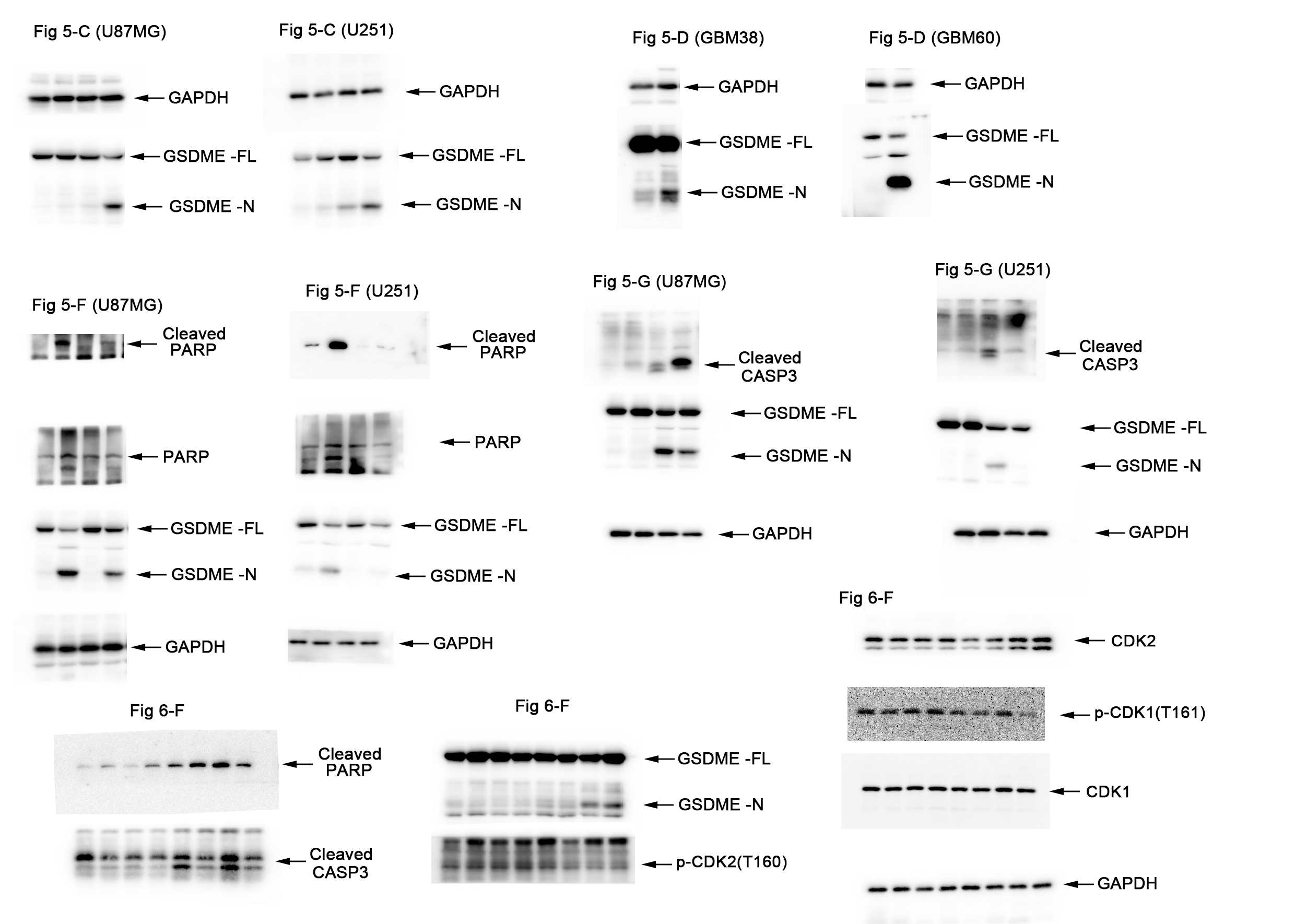

Supplement: Supplementary file 6 — Original Data File [file 41419_2022_5528_MOESM6_ESM.tif]
